# Association between frailty and cognitive status among ambulating Korean elderly: An observational study

**DOI:** 10.1097/MD.0000000000039222

**Published:** 2024-08-09

**Authors:** Sujin Lee, Jae Ho Chung

**Affiliations:** a Department of Neurology, International St. Mary’s Hospital, Catholic Kwandong University College of Medicine, Incheon, Republic of Korea; b Department of Internal Medicine, International St. Mary’s Hospital, Catholic Kwandong University College of Medicine, Incheon, Republic of Korea.

**Keywords:** cognition, elderly, frailty

## Abstract

We aimed to determine the association between frailty and cognitive status of the elderly population in Korea. We examined data from 9920 elders who participated in the 2020 Survey of Living Conditions and Welfare Needs of Korean Older Persons. Frailty was assessed using the Korean version of the Fatigue, Resistance, Ambulation, Illnesses, and Weight Loss scale. The Korean mini-mental status examination was used to test cognitive function. Several logistic regression analysis was performed, with correction for several confounding variables (socioeconomic, health behavior, psychological characteristics, and functional status), to evaluate the relationship between frailty and cognitive state. Of the elderly population in Korea, 1451 (14.6%) were frail and 5977 (60.3%) were pre-frail. Compared to the non-frail group (20.3%), cognitive impairment was considerably higher in the pre-frail (33.1%) and frail (39.8%) groups. When compared to the non-frail group, cognitive impairment was substantially linked to a higher risk of frailty after adjustment (pre-frail odds ratio [OR]: 1.66, 95% confidence interval [CI]: 1.47–1.88; frail OR: 2.00, 95% CI: 1.68–2.37). When cognitive impairment and frailty subcomponents were present, there was a higher likelihood of severe resistance (OR: 1.89; 95% CI: 1.70–2.11) and ambulation (OR: 1.46, 95% CI: 1.32–1.63) issues. Frailty is associated with cognitive impairment.

## 1. Introduction

With aging, body organs’ homeostatic reserves degrade, which is known as frailty.^[1]^ Physical performance decreases and cognitive function and comorbidities increase in accordance with a decline in physiological reserves with age. Frailty eventually develops, leading to functional dependency, worsening of illness, hospitalizations, and an increase in mortality.^[2]^ Many different frailty criteria and indices have been developed. A suitable and practical methodology for identifying frailty in clinical practice is essential for geriatric research. According to Morley et al,^[3]^ the Fatigue, Resistance, Ambulation, Illnesses, and Weight Loss (FRAIL) scale is a simple 5-item questionnaire that can be used to evaluate frailty without the requirement for a physical examination. The Korean version of the scale (K-FRAIL) has been validated in the Korean population.^[4]^ Frailty and cognitive impairment, including dementia and mild cognitive impairment (MCI), have been linked in previous longitudinal and cross-sectional investigations.^[5–7]^ According to the literature, frailty may accelerate cognitive decline and increase the risk of cognitive impairment, including MCI,^[8]^ and frailty may be linked with pathological findings in Alzheimer disease and vascular dementia. These pathophysiological findings point to a common biological pathway between frailty and cognitive impairment.^[9,10]^ However, there are inconsistent findings on the association between frailty and cognitive impairment.^[9,11–13]^ Although Buchman et al^[9]^ demonstrated a significant association, other studies did not find any association.^[11–13]^ To our knowledge, there is no study of the association between cognitive impairment and frailty using the K-FRAIL in a Korean population. Thus, in the present study, we investigated whether there was an association between frailty and cognitive impairment.

## 2. Methods

### 2.1. Study participants

We used data from the 2020 Korea Institute for Health and Social Affairs Survey of Living Conditions and Welfare Needs of Korean Older Persons, a representative national survey of older people in Korea who are not institutionalized. In-person interviews were carried out by skilled interviewers for this survey. Since all participants gave their informed consent and the data was available to the public, no additional ethical approval was needed.^[14]^

There were 10,097 participants in this study who were 65 years of age or older. A total of 177 elderly individuals were eliminated from the study due to their incompleteness of the mini-mental status examination in Korean (MMSE-KC), the K-FRAIL scale, limited activities of daily living (ADL), restricted instrumental activities of daily living (IADL), and the short form of the geriatric depression scale (GDS). Nine thousand nine hundred twenty people (males, n = 3971; females, n = 5949; age range: 65–99 years) made up the final sample. The results of our survey are openly accessible. All participant data were fully anonymized prior to publication. Our work was excluded from Korea’s Enforcement Rule of Bioethics and Safety Act review list since the data were exempt from IRB review.

### 2.2. Assessments

#### 2.2.1. Sociodemographic and health-related characteristics

Investigations were conducted into sociodemographic variables, including gender, age (65–69, 70–74, 75–79, ≥80 years), marriage, educational level, job status, family income, and religion. We modified physical activities for frailty because inactivity may cause a decrease of muscle mass and strength, which may ultimately result in frailty.^[15]^ Exercise of more than 150 minutes per week was considered physical activity.^[15]^ The definition of alcohol usage was “drinking alcohol ≥ two days/week.” For men, consuming 7 standard-sized drinks or more per day, and for women, 5 standard-sized drinks or more per day, was considered heavy alcohol intake.^[16]^ Nutritional screening initiative criteria^[17]^ was used assessing nutritional status. The use of polypharmacy is associated with frailty in older adults. As a result, we modified the dosage of drugs for frailty. The number of chronic illnesses fell into the following categories: 0 to 1 and <2.

#### 2.2.2. Cognitive function

The MMSE-KC, which has a score range of 0 to 30, was used to evaluate cognitive function.^[18]^ In a prior study.^[19]^ Cognitive impairment was defined as the MMSE-KC score being <1.5 standard deviations from old Korean people adjusted for age, sex, and education.

#### 2.2.3. Elderly depression

The Korean version of the GDS was used to characterize depression.^[20]^ The range of GDS scores was 0 to 15. It has been demonstrated that a cutoff score of ≥8 has high sensitivity and specificity for depression.^[21]^

#### 2.2.4. Functional capacity

Investigations were done on ADL, IADL, and visual and auditory impairment. The Korean ADL scale^[22]^ served as the basis for the ADL evaluation. The Korean Instrumental ADL Scale,^[23]^ which was used to define limitations in IADL, was used.

#### 2.2.5. Evaluation of frailty

The K-FRAIL scale was used to define frailty. A 5-item questionnaire called the K-FRAIL scale measures 5 things: tiredness, resistance, ambulation, sickness, and weight loss.^[24]^ Frailty was defined as 3 items on the K-FRAIL scale with a score of >3.

### 2.3. Data analysis

The fundamental features of the research population were described using descriptive statistical techniques, with percentages and figures provided for each variable. A chi-squared test or Fisher exact test was used for categorical variables, and an analysis of variance was used to evaluate clinical characteristics between the participants in the non-frail, pre-frail, and frail groups. Given that there is evidence that a number of factors, including living and home situations, income, family support, education level, loneliness, and depression, can influence frailty,^[25]^ We made adjustments to the data for these factors. All statistical analyses were created using R programming language and a variety of tools. *P* < .005 was considered to indicate statistical significance.

## 3. Results

Table 1 shows the sociodemographic characteristics of the participants. The frail group was characterized by significantly higher numbers of females and older individuals as compared to the non-frail group. Individuals in the frail group were also more likely than those in the non-frail group to live in rural areas, live alone, be unemployed, be less educated, take less exercise, and have more chronic diseases. In addition, poor self-rated subjective health, nutritional risk, medication use, depression, and suicidal ideation were significantly more common in the frail group than in the non-frail group. Differences in functional status variables in the non-frail, pre-frail, and non-frail groups are shown in Table 2. The MMSE-KC score was significantly lower in the frail group (22.6 ± 5.6) than in the non-frail (26.4 ± 4.6) and pre-frail (23.9 ± 5.2) groups, and cognitive impairment was significantly higher in the pre-frail (33.1%) and frail (39.8%) groups than in the non-frail group (20.3%). The rates of comorbidities (visual, auditory, or chewing impairment), independence in ADL, IADL, leg muscle weakness, fall history, and hospitalization in the last year were significantly higher in the frail group than in the pre-frail and non-frail groups. The prevalence and adjusted odds ratios (ORs) for frailty according to cognitive impairment status are presented in Table 3. After adjusting for sociodemographic variables (i.e., age, sex, residence area, religion, marital status, housing status, employment status, BMI, education level, and economic status), health behavioral variables (smoking, alcohol consumption, nutritional status, exercise, and drug use), psychological variables (i.e., health status, stress, depression, social activities, mistreatment of elderly, and suicidal ideation), and comorbidities (visual, auditory, or chewing impairment), and functional status (leg muscle weakness and limitations in ADL or IADL), cognitive impairment was significantly associated with an increased risk of frailty (pre-frail, OR: 1.66; 95% confidence interval [CI]: 1.47–1.88; frail, OR: 2.00, 95% CI: 1.68-–2.37) compared to the non-frail group. The presence of frailty subcomponents, combined with cognitive impairment, was more likely to be associated with severe resistance (OR: 1.89, 95% CI: 1.70-–2.11) and ambulation (OR: 1.46, 95% CI: 1.32-–1.63) difficulties.

**Table 1:** Differences of socio-demographics and clinical characteristics.

	Total	Non-frail (n = 2492)	Pre-frail (n = 5977)	Frail (n = 1451)	*P* value	Post hoc
Sex						
Male	3971 (40.0)	1326 (53.2)	2184 (36.5)	461 (31.8)	<.001	a ^,^ b ^,^ c
Female	5949 (68.2)	1166 (46.8)	3793 (63.5)	990 (68.2)		
Age	73.4 ± 6.5	70.5 ± 5.1	74.1 ± 6.6	75.9 ± 6.9	<.001	a ^,^ b ^,^ c
Age category						
65 to 69	3511 (35.4)	1367 (54.9)	1839 (30.8)	305 (21.0)	<.001	a ^,^ b ^,^ c
70 to 74	2466 (24.9)	591 (23.7)	1515 (25.3)	360 (24.8)		
75 to 79	1956 (19.7)	358 (14.4)	1256 (21.0)	342 (23.6)		
≥80	1987 (20.0)	176 (7.1)	1367 (22.9)	444 (30.6)		
Residence						
Rural	2924 (28.5)	546 (21.9)	1799 (30.1)	479 (33.0)	<.001	a ^,^ b ^,^ c
Urban	7096 (71.5)	1946 (78.1)	4178 (69.9)	972 (67.0)		
Religion						
Yes	5850 (59.0)	1466 (58.8)	3479 (58.2)	905 (62.4)	.015	b ^,^ c
No	4070 (41.0)	1026 (41.2)	2498 (41.8)	546 (37.6)		
Living status						
Live alone	3177 (31.4)	557 (22.4)	1975 (33.0)	585 (40.3)	<.001	a ^,^ b ^,^ c
Live with family	6803 (68.6)	1935 (77.6)	4002 (67.0)	866 (59.7)		
Job						
Employed	3773 (38.0)	1284 (51.5)	2066 (34.6)	423 (29.2)	<.001	a ^,^ b ^,^ c
Unemployed	6147 (62.0)	1208 (48.5)	3911 (65.4)	1028 (70.8)		
BMI	23.6 ± 2.6	23.7 ± 2.3	23.6 ± 2.6	23.4 ± 2.9	<.001	b ^,^ c
BMI category						
Underweight	214 (2.2)	31 (1.2)	136 (2.3)	47 (3.2)	<.001	a ^,^ b ^,^ c
Normal	3815 (38.5)	884 (35.5)	12`6 (38.7)	615 (42.4)		
Overweight	3269 (32.9)	912 (36,6)	1932 (32.3)	416 (28.7)		
Obese	2631 (26.5)	665 (26.7)	1593 (26.7)	373 (25.7)		
Education, years						
0 to 3	1130 (11.4)	98 (3.9)	779 (13.0)	253 (17.4)	<.001	a ^,^ b ^,^ c
4 to 6	3261 (32.9)	597 (24.0)	2089 (35.0)	575 (39.6)		
≥7	5529 (55.7)	1797 (72.1)	3109 (52.0)	623 (42.9)		
Economic status						
1st quartile	2471 (24.9)	357 (14.3)	1640 (27.4)	474 (32.7)	<.001	a ^,^ b ^,^ c
2nd quartile	2486 (25.1)	520 (20.9)	1510 (25.3)	456 (31.4)		
3rd quartile	2484 (25.0)	741 (29.7)	1471 (24.6)	273 (18.8)		
4th quartile	2478 (25.0)	874 (35.1)	1356 (22.7)	248 (17.1)		
Smoking	1089 (11.0)	414 (16.6)	562 (9.4)	113 (7.8)	<.001	a ^,^ b
Alcohol	1397 (14.1)	515 (20.7)	724 (12.1)	158 (10.9)	<.001	a ^,^ b
Regular exercise	3619 (36.5)	1233 (49.5)	2008 (33.6)	378 (26.1)	<.001	a ^,^ b ^,^ c
Chronic illness						
0 to 1	4600 (46.4)	1523 (61.1)	2609 (43.7)	468 (32.3)	<.001	a ^,^ b ^,^ c
≥2	5320 (53.6)	969 (38.9)	3368 (56.3)	983 (67.7)		
Subjective health status						
Good	4940 (49.8)	1756 (70.5)	2719 (45.5)	465 (32.0)	<.001	a ^,^ b ^,^ c
Moderate	3120 (31.5)	551 (22.1)	2064 (34.5)	505 (34.8)		
Bad	1960 (18.8)	185 (7.4)	1194 (20.0)	481 (33.1)		
Nutrition						
Low nutritional risk	8472 (85.4)	2375 (95.3)	5112 (85.5)	985 (67.9)	<.001	a ^,^ b ^,^ c
Moderate nutritional risk	1248 (13.6)	113 (4.5)	808 (13.5)	327 (22.5)		
High nutritional risk	200 (2.0)	4 (0.2)	57 (1.0)	139 (9.6)		
Medication drug number						
0–1	4817 (48.6)	1582 (63.5)	2741 (45.9)	494 (34.0)	<.001	a ^,^ b ^,^ c
≥2	5103 (51.4)	910 (36.5)	3236 (54.1)	957 (66.0)		
GDS (score ≥ 8)*	1412 (14.2)	115 (4.8)	771 (12.9)	526 (36.3)	<.001	a ^,^ b ^,^ c
Mistreatment of elderly	511 (5.2)	125 (5.0)	324 (5.4)	62 (4.3)	.195	
Suicidal idea	187 (1.9)	27 (1.1)	115 (1.9)	45 (3.1)	<.001	a ^,^ b ^,^ c

BMI = body mass index, GDS = geriatric depression scale.

a Non-frail vs pre-frail.

b Pre-frail vs frail.

c Non-frail vs frail.

* The short version of the GDS contains 15 questions, GDS scores range from 0 to 15, with a score ≥ 8 indicating depression.

**Table 2:** Differences of functional status.

	Total (n = 9920)	Non-frail (n = 2492)	Pre-frail (n = 5977)	Frail (n = 1451)	*P* value	Post hoc
MMSE-KC	24.3 ± 5.3	26.4 ± 4.6	23.9 ± 5.2	22.6 ± 5.6	<.001	a ^,^ b ^,^ c
Cognitive impairment						
Normal	6859 (69.1)	1986 (79.7)	4000 (66.9)	873 (60.2)	<.001	a ^,^ b ^,^ c
Impairment	3061 (30.9)	506 (20.3)	1977 (33.1)	578 (39.8)		
Visual impairment	3286 (33.1)	574 (23.0)	2049 (34.3)	663 (45.7)	<.001	a ^,^ b ^,^ c
Hearing impairment	2297 (23.2)	249 (10.0)	1495 (25.0)	553 (38.1)	<.001	a ^,^ b ^,^ c
Chewing impairment	3751 (37.8)	551 (22.1)	2378 (39.8)	822 (56.7)	<.001	a ^,^ b ^,^ c
Activity of daily living (ADL)						
Dependent	421 (4.2)	7 (0.3)	252 (4.2)	162 (11.2)	<.001	a ^,^ b ^,^ c
Independent	9499 (95.9)	2485 (99.7)	5725 (95.8)	1289 (88.8)		
Instrumental ADL (IADL)						
Dependent	1002 (10.1)	44 (1.8)	642 (10.7)	316 (21.8)	<.001	a ^,^ b ^,^ c
Independent	8918 (89.9)	2449 (98.2)	5335 (89.3)	1135 (78.2)		
Leg muscle weakness	2635 (26.6)	100 (4.0)	1804 (30.2)	731 (50.4)	<.001	a ^,^ b ^,^ c
History of fall last year	633 (6.4)	94 (3.8)	392 (6.6)	`47 (10.1)	<.001	a ^,^ b ^,^ c
Hospitalization previous year	648 (6.5)	85 (3.4)	349 (5.8)	214 (14.7)	<.001	a ^,^ b ^,^ c

The ADL scale consists of 7 items addressing dressing, personal hygiene, bathing, showering, self-feeding, toilet hygiene, and transferring. The IADL scale consists of 10 items about grooming, housekeeping, preparing meals, doing laundry, managing medication, keeping track of finances, transport, responding to changes, and ability to use a telephone.

MMSE-KC = mini-mental status examination.

a Non-frail vs pre-frail.

b Pre-frail vs frail.

c Non-frail vs frail.

**Table 3:** Adjusted odds ratio of cognitive impairment for frailty.

	OR (95% confidence interval)
Frailty	
Non-frail	Reference
Pre-frail	1.66 (1.47–1.88)
Frail	2.00 (1.68–2.37)
Frailty phenotype components	
Fatigue	1.00 (0.90–1.12)
Resistance	1.89 (1.70–2.11)
Ambulation	1.46 (1.32–1.63)
Illness	0.52 (0.21–1.31)
Weight loss	0.96 (0.70–1.33)

OR = odd ratios.

Adjusted for sociodemographic variables (i.e., age, sex, residence area, religion, spouse, living status, job, BMI, education level, economic status), health behavioral variables (i.e., smoking, alcohol, nutritional status, regular exercise and drug), psychological variables (i.e., health status, stress, depression, mistreatment of elderly and suicidal idea), comorbidities, and functional status (visual, hearing, chewing impairment, ADL, IADL, and leg muscle weakness).

## 4. Discussion

In our large sample of community-living Korean elders, using the K-FRAIL scale, we found an association between frailty status and cognitive status. Although comparing the findings of our study with those in the literature is difficult due to differences in frailty definitions, numerous observational studies have found a temporal link between frailty and cognitive impairment. Boyle et al^[8]^ discovered that physical frailty among older persons who had no cognitive impairment at the start of the study was associated with an increased probability of developing MCI over the next 12 years. Our findings contribute to the current understanding of the connection between frailty and cognitive impairment by demonstrating that frailty is unaffected by many confounding variables. Meta-analyses and other observational studies^[19,26]^ have indicated a strong link between frailty and the likelihood of cognitive impairment, regardless of other risk variables or frailty scale utilized. However, previous studies on the association between frailty status and cognitive impairment produced inconclusive results.^[9,11–13]^ Of these 4 studies, only one of the studies^[9]^ found a significant positive association, with the other studies finding a negative association. The pathophysiological mechanism of cognitive impairment in frailty is not clear. However, both frailty and cognitive impairment share the same risk factors and clinical manifestations and may even share a common pathological basis.^[9,10,13]^ Given the frequency with which frailty and cognitive impairment co-occur in the elderly, it is possible that cognitive impairment is a component of frailty. It is conceivable that abnormal brain alterations or other underlying pathology could be the cause of both dementia and frailty.^[27]^ A recent study from the Rush Memory and Aging Project and Religious Orders Study found a correlation between brain pathology and frailty in the years before death in Alzheimer disease patients based on longitudinal frailty data and autopsy results related to brain pathology.^[23]^ Another view is that cognitive impairment is a component of frailty, rather than 2 distinct disease entities that regularly coexist in old life. Many specialists believe that cognitive function should be added as one of the frailty criteria. The International Academy on Nutrition and Aging and the International Association of Gerontology and Geriatrics have proposed a new term, “cognitive frailty,” to describe a heterogeneous clinical state characterized by both physical frailty and cognitive impairment without Alzheimer disease or other forms of dementia.^[24]^ According to cognitive frailty theory, subclinical cognitive pathophysiology may be a component of frailty that is manifested years before overt dementia develops and frailty precedes and predicts the development of dementia. In a previous study, the authors found no significant associations between frailty status and dementia and no dose-response relationship in risk measures according to frailty status in a cohort or in subgroups with and without cognitive impairment.^[28]^ These findings suggest that frailty and cognitive impairment are separate processes.^[28]^ If frailty and cognitive impairment/dementia are distinct entities, their interactions may be bidirectional, exacerbating each other in a vicious cycle. Evidence for the latter has been found in several cross-sectional and longitudinal studies, which showed that individuals with dementia or cognitive impairment were more likely to be frail or become frail.^[29]^ Although frailty is important for health outcomes, as it is associated with poor quality of life and an increased risk of mortality, assessing frailty in clinical practice using current frailty criteria is time consuming and difficult. Due to its simplicity and speed of administration, the K-FRAIL scale can be used to quickly assess frailty in the clinical setting both by physicians and patients. Thus, the K-FRAIL scale might be a useful screening tool for physicians in busy practices worldwide, including those in Korea. Our study has some limitations. First, due to our cross-sectional study design, it is not possible to rule out a cause-and effect relationship between frailty status and cognitive impairment. Second, while the MMSE is the most widely used screening tool for measuring cognitive impairment, it has little utility for diagnosing moderate cognitive impairment.^[30]^ Although above positive advantages, there is no psychotherapy and counseling intervention data in our survey. This is our last survey limitation.

In conclusion, our research showed a correlation between cognitive impairment and frailty status. This finding suggests that more treatments are needed to lessen the burden of frailty and cognitive impairment in the elderly. Preventive interventions to delay or slow down cognitive deterioration and frailty have not yet been the subject of any prospective research. Strategies for managing frailty that are appropriate, such rehabilitation, may help to prevent cognitive impairment. To demonstrate links between frailty and cognitive decline in the senior population, more carefully planned prospective studies are required.

## Author contributions

**Conceptualization:** Sujin Lee.

**Formal analysis:** Sujin Lee, Jaeho Chung.
